# Advances in Dairy Cattle Reproduction—A Foreword

**DOI:** 10.3390/ani14182650

**Published:** 2024-09-12

**Authors:** Fernando López-Gatius

**Affiliations:** 1 Agrotecnio Centre, 25198 Lleida, Spain; lopezgatiusf@gmail.com; 2 Subunit, Transfer in Bovine Reproduction SLu, 22300 Barbastro, Spain

Dairy cattle have played an important role in economic development since the beginning of agriculture. In essence, dairy cattle herding had already become a dominant part of the culture and economic development during the Neolithic era [1]. Subsequently, man–cow interactions have been multifaceted, from a genetic mutation that gave people the ability to produce lactase and drink milk throughout their lives [2] to the production of a vaccine against Variola (Smallpox virus) [3]. It is equally important that reproductive technologies developed for dairy cattle provide a spin-off in mammalian species [4]. Animal breeding and assisted reproduction in women were revolutionized worldwide during the 20th century by discoveries on spermatozoa preservation, oocyte maturation, embryo transfer, and embryo freezing in cattle. For example, Dr. G. Pincus and Dr. M.C. Chang were students at the Cambridge Animal Research Station before developing the contraceptive pill and pioneering fertilization studies [5]. Currently, we share concerns over global warning and the impacts of the COVID-19 crisis. In this context, aspects of the reproduction control of cattle seem to have plateaued. However, changes in both genetic screening to increase fertility and management practices to promote cow comfort have recently improved reproductive efficiency [6,7]. In addition, by reducing the incidence of reproductive disorders such as anestrus, low fertility, pregnancy failure, or perinatal mortality that are essential to the health and welfare of cows, the use of land resources will be more efficient [7,8,9]. In fact, cow health and welfare have been extensively associated with higher milk production alongside high fertility in the past two decades [6,7,10,11]. Therefore, it is a good time to emphasize the importance of effective reproductive management, which not only benefits the economics of herds but also reduces greenhouse gas emissions [7,12,13]. This lowers public concern about dairy production [7,8,14,15]. The thirteen articles (seven original articles, four reviews, and two commentaries) included in the Special Issue entitled “Advances in Dairy Cattle Reproduction” have thus sought to better understand and monitor the reproductive physiology of the dairy cow. These have been published in recent issues of Animals and are compiled on the journal’s webpage: https://www.mdpi.com/journal/animals/special_issues/frontiers_in_dairy_cattle_reproduction.

The term advance might suggest that there is a difficult borderline in dairy cattle reproduction. However, knowledge transfer on the reproductive management of dairy cows is enormous. In effect, every detail within a herd’s routine can be improved so that focus on cutting-edge technology is addressed by all authors in the Special Issue. The establishment of pregnancy to term remains the primary goal in most dairy systems [7,15,16]. This means that clinical and practical approaches from different fields may be a source of applications to improve reproductive efficiency in herds. Thus, several authors describe strategies to improve reproductive parameters. Important economic losses are linked to the repeat breeder cow syndrome worldwide. Carlos Carmelo Pérez-Marín and Luis Angel Quintela [17] compile information about recent knowledge on the pathogenesis of this syndrome and discuss potential therapies and management strategies to reduce its incidence in herds. Ottó Szenci [18] documents how the negative effect of dystocia on calves and their dams can be reduced using different precision farming devices to predict the onset of parturition. Silviu-Ionut Bors and Alina Bors [19] show that the profitability of dairy farms can be improved by rebreeding the nonpregnant cows after early negative pregnancy diagnosis (25 days after artificial insemination (AI)). Zoltán Szelényi et al. [20] propose to reduce the incidence of retained placenta and of a large uterus at AI to improve the rate of pregnancy maintenance. Scoring the size and position of the pre-breeding uterus may provide a useful source of information to manage fertility in dairy cows [21,22,23]. There is much to be learned about factors and their interactions influencing pregnancy loss of non-infectious causes after a positive pregnancy diagnosis (during the late embryonic/early fetal period) [16,24,25]. Even the clinician himself can be a risk factor for pregnancy loss [25]. Fernando López-Gatius [26] outlines that therapeutic abortion using an increased prostaglandin F2α (PGF2α) dose offers benefits in cows carrying dead twins during the early fetal period (49–55 days post-AI). Indeed, the increase in the standard dose of the PGF2α or their analogs to induce luteolysis is beneficial under certain circumstances in high-producing dairy cows [27]. The use of sex-sorted semen has increased sharply over the last decade, but it is significantly less fertile than conventional semen [28,29,30]. This is the reason why the timing of AI was revisited, proposing the end of estrus rather than its onset as the best guide for AI timing in dairy cattle undergoing spontaneous estrus [31]. Using the dairy cow as a model, Olimpia Barbato and colleagues [32] update the use of pregnancy-associated glycoprotein (PAG) measurements to improve reproductive management in bovine as well as in small ruminants and buffalo. The authors conclude that PAG measurements using on-farm pregnancy tests are a feasible method of pregnancy confirmation at herd level [32].

Reproduction is a physiological function very sensitive to disruption by multiple stressors, particularly when mammals are under heat stress conditions [33,34]. Perturbations in follicular physiology during a period of thermal stress are delineated by Fabio De Rensis and partners [35]. Oocytes, which are also very sensitive to any type of stress [36], carry the intercellular communication in the mammalian ovary, including follicular development and formation of the follicular fluid [37,38]. This is of relevance to selecting valuable oocytes/follicles for assisted reproductive technologies. Since 2017, due to the increasing availability and lower costs of production, the global use of in vitro-produced (IVP) embryos has surpassed the number of in vivo-derived embryos [39]. However, the technical aspects of IVP embryos remain to be optimized [40,41,42]. Olga Witkowska-Piłaszewicz and colleagues [43] expand knowledge associated with follicle health evaluation as potential applications for assisted reproduction procedures. Using Brilliant Cresyl Blue staining, Heinrich Bollwein and colleagues [44] provide a further oocyte quality description to select the best oocytes when damaged sperm have to be used in in vitro fertilization processes. Finally, three papers on reproductive genomics provide some basis for both research and resource optimization in the dairy sector. In this time of advances in reproductive genomics [45], mechanisms involved in corpus luteum formation, function, and regression must be better understood, particularly in high milk producers. Indeed, the concept of luteal deficiency, which was already defined in 1949 [46], has been widely developed in humans [47,48]. However, clinical manifestations of sub-luteal function in cattle, which as in the human species result in low fertility [17] and early pregnancy loss [16], have not received widespread consideration. Findings by Michael W. Pfaffl and colleagues [49] reveal a clear involvement of adipokines in the local mechanisms of luteal function. Darren K. Griffin’s group [50], working with the bull (dairy and beef), presents the incidence and potential costs (financial and environmental) of genetic abnormalities. Fernando A. Di Croce’s group [51], working with Holstein dairy cows, proves that the inclusion of cow abortion genomic predictions in a multi-trait selection index would allow dairy producers to reduce the incidence of abortion and to select high-producing, healthier, and more profitable cows.

For that matter, we believe there is much to be learned by exploring all the articles in the Special Issue. We hope that these new insights will lead to a better understanding of the reproductive function of the cow at the farm level and to the development of new strategies to improve it. Focus on physiological functions important for reproduction will improve the health and welfare of the dairy cow, favoring both increased productivity and a reduction in the carbon footprint of dairy production systems.
